# Muscarinic acetylcholine receptors are expressed by most parvalbumin-immunoreactive neurons in area MT of the macaque

**DOI:** 10.1002/brb3.225

**Published:** 2014-03-21

**Authors:** Anita A Disney, Hussein A Alasady, John H Reynolds

**Affiliations:** Systems Neurobiology Laboratories, The Salk Institute for Biological StudiesLa Jolla, California

**Keywords:** Anatomy, calcium-binding proteins, immunofluorescence, neuromodulation, parvalbumin, quantitative, visual cortex

## Abstract

**Background:**

In the mammalian neocortex, cells that express parvalbumin (PV neurons) comprise a dominant class of inhibitory neuron that substantially overlaps with the fast/narrow-spiking physiological phenotype. Attention has pronounced effects on narrow-spiking neurons in the extrastriate cortex of macaques, and more consistently so than on their broad-spiking neighbors. Cortical neuromodulation by acetylcholine (ACh) is a candidate mechanism for aspects of attention and in the primary visual cortex (V1) of the macaque, receptors for ACh (AChRs) are strongly expressed by inhibitory neurons. In particular, most PV neurons in macaque V1 express m1 muscarinic AChRs and exogenously applied ACh can cause the release of *γ*-aminobutyric acid. In contrast, few PV neurons in rat V1 express m1 AChRs. While this could be a species difference, it has also been argued that macaque V1 is anatomically unique when compared with other cortical areas in macaques.

**Aims:**

The aim of this study was to better understand the extent to which V1 offers a suitable model circuit for cholinergic anatomy in the macaque occipital lobe, and to explore cholinergic modulation as a biological basis for the changes in circuit behavior seen with attention.

**Materials and methods:**

We compared expression of m1 AChRs by PV neurons between area V1 and the middle temporal visual area (MT) in macaque monkeys using dual-immunofluorescence confocal microscopy.

**Results and conclusion:**

We find that, as in V1, most PV neurons in MT express m1 AChRs but, unlike in V1, it appears that so do most excitatory neurons. This provides support for V1 as a model of cholinergic modulation of inhibition in macaque visual cortex, but not of cholinergic modulation of visual cortical circuits in general. We also propose that ACh acting via m1 AChRs is a candidate underlying mechanism for the strong effects of attention on narrow-spiking neurons observed in behaving animals.

## Introduction

The cortical neuromodulator acetylcholine (ACh) has been implicated in diverse brain processes, both normal and pathological (Bakin and Weinberger 1996; Everitt and Robbins 1997; Nobili and Sannita 1997; Hyde and Crook 2001; Maskos et al. 2005; Sarter et al. 2005). In particular, in studies of the rodent cortex, both in vivo and in vitro*,* phasic release of ACh has been linked to attentive states (Sarter et al. 2005). Interactions between cholinergic activity and attention have also been reported in the primary visual cortex (striate cortex, V1) of the behaving macaque monkey (Herrero et al. 2008).

In the behaving macaque, it is known that the effects of attention on spike rate in extrastriate area V4 are strong and highly consistent in a population of neurons that exhibit narrow spikes, but do not produce those spikes in bursts (Mitchell et al. 2007; Anderson et al. 2011a). These narrow-spiking, nonbursting neurons are likely to correspond largely to the immunocytochemically-defined population of parvalbumin-immunoreactive (PV) inhibitory neurons (Kawaguchi and Kubota 1993; Chow et al. 1999; Constantinople et al. 2009; Anderson et al. 2011a). We have shown that in macaque V1, muscarinic ACh receptors (AChRs) are strongly expressed by inhibitory interneurons (Disney et al. 2006, 2007) and in particular that at least 75% of PV neurons express m1-type muscarinic AChRs (Disney and Aoki 2008). In contrast, in rat V1 only 27% of neurons that express PV also express m1 AChRs (Disney and Reynolds 2014).

While this differing expression of muscarinic AChRs by PV neurons in rat versus macaque V1 may reflect a species difference, macaque V1 differs in some ways from other cortical areas in the macaque. For instance, while 25% of neurons across most of macaque cortex are inhibitory (Hendry et al. 1987), inhibitory neurons comprise only 20% of neurons in macaque V1 (Hendry et al. 1987; Beaulieu et al. 1992) and the subtype composition of this inhibitory population differs from that in nearby visual cortical areas (DeFelipe et al. 1999). Similarly, while 50% of GABAergic neurons in the prefrontal cortex of macaques (Conde et al. 1994) and in V1 of rats (Gonchar and Burkhalter 1997) express PV, in macaque V1 PV neurons comprise 74% of the GABAergic population (Van Brederode et al. 1990). Thus it is not necessarily appropriate to assume that anatomical data on AChR expression gathered in macaque V1 can be applied in attempting to understand the cholinergic modulation of macaque cortex in general or as the basis for proposed mechanisms underlying the effects of attention (or other behavioral phenomena) in extrastriate visual areas.

We examined whether PV neurons in extrastriate area middle temporal (MT) express m1-type muscarinic AChRs; the class of ACh receptor most frequently expressed by PV neurons in area V1. m1 AChRs are a likely mediating receptor type if cholinergic mechanisms are to be considered a candidate explanation for attention-related spike rate increases among narrow-spiking neurons in the extrastriate cortex. Another possible mediator would be the homomeric *α*7 subunit-containing nicotinic receptor. Unfortunately antibodies directed against the *α*7 nicotinic receptor subunit did not pass our controls for use in macaque neocortex and so this important receptor class was not examined in this study. High affinity (heteromeric) nicotinic receptors, on the other hand, are not strongly enough expressed in the occipital lobe of macaques outside layer 4c of V1 (Disney et al. 2007) to be a candidate for attention-related changes in spiking activity in area MT. And finally, the other prominent class of cortical muscarinic receptor – the m2-type AChR – would not be expected to increase spike rate specifically in PV neurons, as it is usually axonally expressed (Mrzljak et al. 1993; Brown et al. 1997; Disney et al. 2006, 2012) and typically acts to reduce GABA release when expressed by PV neurons (Kruglikov and Rudy 2008). Thus, to be a receptor underlying increased narrow-spiking neuron firing in response to ACh, m2 AChRs would have to be specifically localized at synapses onto other PV neurons and not onto other cell classes, which has not been reported (Mrzljak et al. 1993; Disney et al. 2006, 2012).

We find that m1 AChRs are equally strongly expressed by PV neurons in macaque area MT as they are in macaque V1. This lends support to the idea that cholinergic modulation of PV neuron-mediated inhibition is a general feature of visual processing in primates (Disney and Reynolds 2014). We also find that more non-PV neurons express m1 AChRs in MT than in V1. The implications of these data for ACh as a candidate mechanism that supports attentive states is discussed in the context of likely downstream targets for m1 AChR in various cell classes and in different species.

## Materials and Methods

### Histological preparation

Three adult male macaque monkeys (two *Macaca mulatta* and one *Macaca nemestrina*) that had previously been used in unrelated electrophysiology recordings were used in this experiment. Tissue was obtained from the unrecorded hemispheres. For further details of the standard protocols for the donor labs, see Oristaglio et al. (2006) and Nauhaus et al. (2012). All procedures were approved and performed in accordance with NIH and institutional guidelines for the care and use of animals.

Animals were euthanized by intravenous injection of sodium pentobarbital (60 mg/kg). Following complete abolition of corneal and pedal reflexes, animals were transcardially perfused with heparinized 0.01 mol/L phosphate-buffered saline (PBS, pH 7.4) followed by 4 L of chilled 4% paraformaldehyde (PFA) in 0.1 mol/L phosphate buffer (PB, pH 7.4). The fixative was run for at least 40 min. The brain was then removed and blocked as necessary to provide donor labs with tissue for their histological needs. The remaining tissue was post-fixed overnight at 4°C in 4% PFA. The following day, the brain was transferred to 30% sucrose in PBS as a cryoprotectant and stored at 4°C until it sank.

Hemispheres to be sectioned were blocked in approximately the coronal plane at the level of the lunate sulcus (with the whole lunate sulcus in the block) and at the anterior tip of the intraparietal sulcus. The tissue between these two blocking cuts was sectioned at a thickness of 50 *μ*m on a freezing microtome. To provide reference sections for determining boundaries between cortical areas and cortical layers, two 1-in-6 series were set aside; one for Gallyas (Gallyas 1970) and the other for Nissl (cresyl violet) staining. The remaining sections were stored at 4°C in PBS with 0.05% sodium azide added.

### Source and characteristics of primary antibodies

Please see Table 1 for a summary of the antibodies used in this study.

**Table 1:** Primary antibodies

Antigen	Immunogen	Manufacturer, species, clonality, catalog and lot numbers	Dilution
m1 muscarinic acetylcholine receptor	GST fusion protein corresponding to aa227-353 of human m1 ACh Receptor (GSETPGKGGGSSSSSERSQPGAEG SPETPPGRCCRCCRAPRLLQAYSW KEEEEEDEGSMESLTSSEGEEPGES VVIKMPMVDPEAQAPTKQPPRSSP NTVKRPTKKGRDRAGKGQKPRGK EQLAKRK)	Alomone Labs (Jerusalem, Israel). Rabbit polyclonal. Cat#AMR-001 Lot#AN-05	1:1000
Parvalbumin	Purified carp parvalbumin	Swant (Bellinzona, Switzerland). Mouse monoclonal. Cat#235 Lot#10-11(F)	1:1000

We detected m1 muscarinic ACh receptors (m1 AChRs) using a polyclonal antibody raised in rabbit against amino acids 227–353 of the intracellular loop i3 of the human m1 AChR, obtained from Alomone Labs (Jerusalem, Israel, catalog #AMR-001, lot # AN-05). This region of the i3 loop has high sequence homology (99%) with the macaque m1 AChR.

To detect parvalbumin (PV) we used a monoclonal antibody produced by hybridization of mouse myeloma cells with spleen cells from mice immunized with parvalbumin purified from carp muscles (Swant, Bellinzona, Switzerland, catalog #235, lot#10-11[F]).

The antibody used to detect parvalbumin has been characterized by immunoblot and radioimmunoassay on tissue homogenates (Celio et al. 1988) and does not stain tissue from the brains of parvalbumin knockout mice (Schwaller et al. 1999). Additional control experiments for use of both antibodies in macaque tissue are described under Antibody controls below.

### Immunocytochemistry

A 1-in-24 series (1.2 mm between processed sections) with a random starting well was taken from the set of all tissue sections cut for each animal. The set from which tissue was drawn covered the region of brain between the lunate sulcus and the anterior tip of the intraparietal sulcus. This resulted in at least one (usually two) tissue sections being processed per animal that contained both area MT and area V1 and a third (more anterior) section that contained MT only. All sections in the 1-in-24 series were processed, but data were collected from these two or three MT-containing sections.

Tissue sections were pre-incubated in a blocking buffer comprising 1% IgG-free bovine serum albumin (BSA; Jackson ImmunoResearch, West Grove, PA), 0.05% sodium azide (Sigma, St. Louis, MO), 0.5% Triton X-100 (Sigma), and 5% normal donkey serum (Jackson ImmunoResearch) in PBS for 60 min before being transferred into fresh blocking buffer with primary antibodies added. Free-floating sections were usually exposed to antibodies directed against PV (1:1000) and m1 AChRs (1:1000) in a single co-incubation step. In a single processing batch, the antibodies were applied in separate incubation steps. Results did not differ depending on whether co-incubation or separate incubations were used and the data were combined.

The tissue remained in the antibody solution for 24–72 h on a shaker at room temperature. After rinsing thoroughly with PBS, the tissue was transferred into diluted secondary antibodies (1:200 in PBS with 1% BSA). Both secondary antibodies were raised in donkey. PV-immunoreactive sites were visualized using the DyLight 488 nm fluorophore (DyLight 488 donkey anti-mouse IgG; Jackson ImmunoResearch, cat# 715-486-150, lot # 95844). m1 AChR-immunoreactive sites were visualized using the DyLight 594 nm fluorophore (DyLight 594 donkey anti-rabbit IgG; Jackson ImmunoResearch, cat# 711-516-152, lot # 97356). This second incubation proceeded in the dark, on a shaker at room temperature, for 4–6 h.

The sections were rinsed in PBS, mounted, and dried overnight in the dark. They then underwent dehydration through a graded series of alcohols (50–100%), followed by 2 × 100% xylene, and were coverslipped with DPX mounting medium (Electron Microscopy Services). Slides were stored at room temperature in light-protective boxes.

### Antibody controls

#### Primary antibodies

Antibodies directed against the epitope we used to localize m1 AChRs label a single band at ∼78 kD in Western blots of homogenate from macaque V1 (Disney et al. 2006). To further rule out nonspecific binding, preadsorption controls were performed for both of the primary antibodies used in this study. Effectiveness of preadsorption was assessed by immunoperoxidase detection (Hsu et al. 1981). A control peptide representing the same amino acid sequence as was used in production of the m1 AChR antibody (a.a. 227-353 of human m1 AChR) was provided with the antibody by the manufacturer (Alomone Labs, Jerusalem, lot AN-05). Recombinant rat parvalbumin (produced in *Escherichia coli*) was purchased from Swant (lot# 5.'93). Antigens were diluted at 50 nmol/L (m1 antigen) and 100 nmol/L (parvalbumin) in a premixed antibody solution (in both cases the antibodies were diluted to 1:1000). The antibody-antigen solution was set on a shaker at room temperature for 2–3 h. The preadsorbed antibody was then used (as-is with no spin-down or filtration step) in the following manner.

After blocking steps for endogenous peroxidase activity (30 min in 1% hydrogen peroxide in PBS) and protein-protein interactions (60 min in PBS with 1% BSA, 5% normal goat serum, .05% sodium azide, 0.5% Triton X-100) two sections (co-incubated to this point) from the same animal were separated. One was placed in the preadsorbed antibody solution and the other in a regular antibody solution (1:1000). After overnight incubation at room temperature on a shaker, and thorough rinsing, the sections were placed in biotinylated secondary antibodies (biotin conjugated goat anti-rabbit IgG, cat#111-066-003, lot#70900, or biotin-conjugated goat anti-mouse IgG,; cat#115-066-003, lot#76905, both from Jackson ImmunoResearch) diluted at 1:1000 in PBS with 1% BSA added. After 1 h in this solution at room temperature on a shaker, the sections were rinsed and incubated for 30 min in an avidin-horseradish peroxidase complex (Vector Elite ABC Kit, Vector Labs, Burlingame, CA). Staining was visualized using the Vector VIP kit (Vector labs). The tissue exposed to the regular antibody solution (i.e., not preadsorbed) was reacted first and the development time needed to clearly visualize staining was determined (usually 2–4 min). The tissue exposed to the preadsorbed antibody was then reacted for the same duration in fresh VIP solution. Preadsorption eliminated staining for both m1 AChRs and for parvalbumin, while normal staining was seen in tissue sections simultaneously processed using antibodies that had not been preadsorbed.

#### Secondary antibodies

To confirm the specificity of the secondary antibodies, tissue sections were incubated in blocking solution without primary antibodies added (no primary control). In these controls, tissue sections were incubated overnight in blocking solution only and then processed according to the regular protocol, as described above. This processing resulted in no fluorescent signal.

We also conducted a control experiment in which we incubated tissue sections, which had been exposed to a primary antibody, in a solution containing a mismatched secondary control (a secondary antibody that targeted a different species than the host animal from which the primary antibody was derived, e.g., tissue exposed to the rabbit anti-m1 AChR primary antibody and then the donkey anti-mouse IgG secondary antibody). This incubation also produced no fluorescent signal, indicating no cross-reactivity of the secondary antibodies with the nontarget primary antibody.

### Locating areas MT and V1

**Figure 1:**
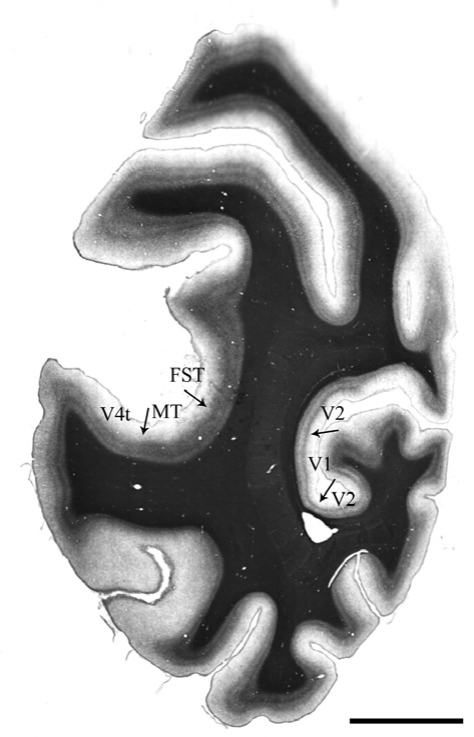
Myelin stained tissue showing areas middle temporal (MT) and V1 in the same tissue section from animal A4. Arrows show the approximate location of the architectonic boundaries between visual areas, as determined from the pattern of myelin staining (Gallyas, see Methods). MT, V1 and the immediately adjacent areas (V4t, FST, and V2) are shown. Scale bar = 5 mm.

Using Gallyas reference sections immediately adjacent to the dually-labeled (data) sections, area MT was identified based on its location within the superior temporal sulcus and its dense, matted myeloarchitecture (Fig. 1). Coregistration of the reference and data sections was achieved using gross morphology, pial surface shape, cutting and other artefacts and blood vessels as fiduciary marks. The location of the borders of MT were physically drawn onto the coverslip of the data sections with a ±1000 micron confidence boundary defined before imaging. Area V1 was identified using the Stria of Gennari, which is clearly visible directly on the sections.

### Confocal microscopy and cell counting

The “Tile Scan” function on a Zeiss LSM780 laser scanning confocal microscope was used for data collection. The 488 nm and 561 laser lines were used for fluorophore excitation. Laser power was chosen independently for each line such that with a given line turned off, no image was captured in the corresponding data channel. Typically the 488 line was used at 0.7% power and the 561 line at 1% power. These bleed-through checks were done when switching between animals or between tissue samples processed in different batches. The pinhole was set to 34 *μ*m and data for both channels collected concurrently.

Area V1 (if present) was found at 10× magnification based on gross section morphology and the presence of the Stria of Gennari. A “*z*-stack” was then collected at 40× magnification (water immersion), just below the pia and spanning the entire tissue thickness to determine antibody penetration and appropriate imaging depth. A second *z*-stack was then collected in the same cortical column, just above the white matter. These stacks were used to select a single imaging plane that could be used to scan a 223 micron-wide column of tissue spanning the entire cortical thickness from pia to white matter. A wide-field (usually >650 micron) overview scan was used in coregistering the data scan with reference sections, allowing identification of layer boundaries (see below). The individual “tiles” of each pia-to-white matter scan were stitched using the Zeiss Zen software (2010; Carl Zeiss Microscopy, LLC, Thornwood, NY). Both the raw and stitched scans (as well as the *z*-stacks) were saved for offline analysis. This process was repeated for area MT.

#### Determining layer boundaries

For each immunolabeled section, layer boundaries were determined using Nissl reference sections. Digital images of the Nissl sections were taken with a 25× objective on a Zeiss Axio Observer VivaTome microscope. Co-registration of the fluorescence and light microscopic images was achieved using gross morphology, pial surface shape, cutting and other artefacts, and blood vessels as fiduciary marks. The depths *–* in microns from the pial surface *–* of layers 4a, 4b, 4c, 5, and 6 (V1), or layers 4, 5, and 6 (MT) were recorded on the reference images. These measurements were then converted to the magnification of the data images and the layer boundaries drawn with a ±10 *μ*m confidence boundary onto TIFF image files using Photoshop (Adobe). The depth of the boundary between layers 1 and 2 was determined by eye, based on the sharp increase in the density of cell somata at the layer transition.

#### Counting cells

Once the layer boundaries had been drawn, counting was done from TIFF image files using custom software written in Matlab (Mathworks, Natick, MA). Data channels (red and green) were isolated and immunopositive somata counted in each channel separately from gray-scale images. Only wholly visible, in focus, immunolabeled somata were counted. Somata that crossed the left image boundary or the 20 *μ*m confidence boundary around layer borders were excluded, as were objects smaller than 5 *μ*m along their long axis. The *x* and *y* co-ordinates of the center of the cell body were recorded manually. Quantification of single and dual labeling was made from small shapes (equivalent to a five micron object) centered at these *x*/*y* co-ordinates in a new image frame, i.e., in the same frame size as the original TIFF image, but with the data channels turned off. The counting objects had to overlap to be considered dually labeled. In cases where the markings touched but did not overlap, the data channels were inspected and a qualitative determination was made. Roughly 0.5% of the sample required this additional step.

### Qualitative data collection

Qualitative observations were made from the same data images used for quantitative data collection. In describing this “neuropil” (i.e., nonsomatic) staining, we classified the neuropil immunoreactivity as dendritic, axonal, or punctate. Varicose processes with collaterals emerging at right angles were classified as axonal. Dendrites were identified as larger caliber processes of a slightly varicose or nonvaricose nature (i.e., not characterized by the classic “beads-on-a-string” appearance of axons), from which branches emerged at angles of less than 90°. In addition to labeled somata, dendrites, and axons; we defined as “puncta” small spots – approximately 1 micron in diameter or less – that were not visibly attached to any process. These puncta could represent spines, axon terminals, or “islands” of immunoreactivity along larger structure such as a dendrite or axon.

#### Photomicrograph production

Confocal images were captured using the Zeiss Zen 2010 software. Brightness and contrast settings were chosen such that the lumen of any visible vasculature appeared black and to minimize saturation. *γ* correction was not used. Unless noted in the figure legend, the only alterations made for publication were to convert the red/green data images to magenta/green.

#### Analysis

This study was not stereological by design, so the Abercrombie correction (T/T+h: see (Guillery 2002) was applied to reduce the counting bias associated with soma size. Object height (h) was measured along the long axis of the cell soma for a random sample of ten neurons across all layers from at least two tissue sections per monkey, per area. Mean values are listed for each cell type in Table 2.

**Table 2:** Mean soma size (in *μ*m) by cell-type

	V1	MT
PV-ir	13.08 (2.46)	13.86 (2.89)
m1-ir	13.12 (2.96)	13.08 (2.46)

Values in parentheses are the standard deviation of the mean. Number of neurons in each cell: PV in V1 = 61; PV in MT = 67; m1 in V1 = 102; m1 in MT = 97.

To determine a value for T, the mean dehydrated thickness of the tissue was measured as the distance between the upper- and lower-most in-focus planes of the *z*-stacks taken at the beginning of each scan (see Confocal microscopy, above). The obtained value for T (measured across 2–4 sections per animal) did not differ between MT (mean 34 *μ*m, SD 3.77) and V1 (mean 31.25, SD 3.01). The mean thickness collapsed across all three animals and both cortical areas was 32.78 *μ*m (SD 3.64).

The resulting Abercrombie correction factor for both neuronal types in both cortical areas was 0.7. Both raw and corrected counts are reported in the text, all percentages are calculated based on the Abercrombie corrected counts.

## Results

**Figure 2:**
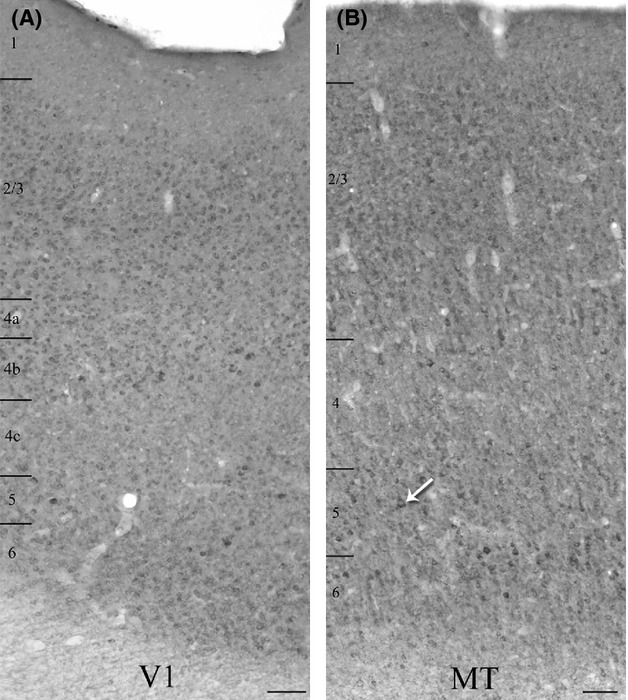
Qualitative comparison of single-label immunoperoxidase reactivity for m1 ACh receptors in V1 (A), and the middle temporal visual area (MT) (B). Immunopositive somata are present in all cortical layers of both areas. Higher levels of neuropil immunoreactivity in area MT (B) make the immunopositive somata more difficult to identify at this magnification (10×) although some strongly labeled cell bodies are evident in layer 5 (see arrow, e.g.). The micrographs present images of the two areas from the same tissue section. Layer boundaries are indicated on the left of each panel. Imaged with a 10× objective. Scale bars = 100 *μ*m.

**Figure 3:**
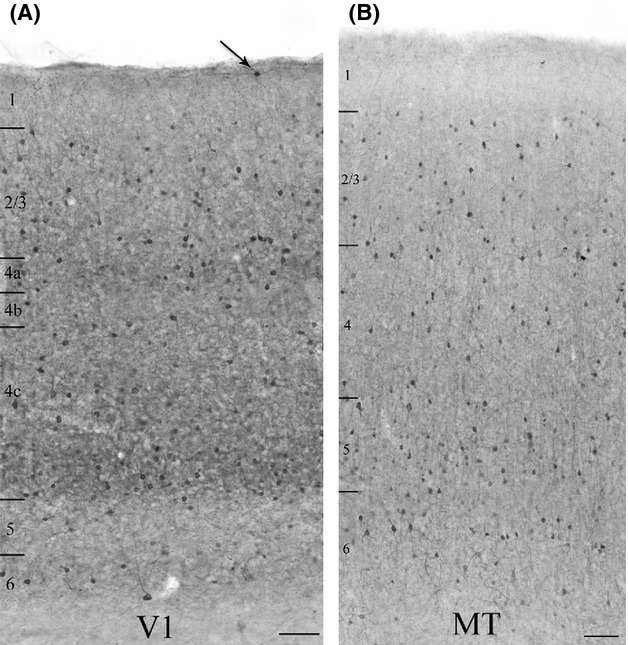
Qualitative comparison of single-label immunoperoxidase reactivity for parvalbumin in V1 (A), and the middle temporal visual area (MT) (B). Parvalbumin (PV) neurons are present in cortical layers 2 through 6 in both areas. In V1 there are occasional PV neurons in layer 1 as well (arrow). There is denser staining of the neuropil and an apparently higher density of somata in the thalamic recipient layers of V1 (layers 4a, 4c, and 6). There is no comparable banding in MT, where the neuropil staining is generally more diffuse. The micrographs present images of different, but coprocessed tissue sections from a single animal. Layer boundaries are indicated on the left of each panel. Imaged with a 10× objective. Scale bars = 100 *μ*m.

We used dual immunofluorescence to determine the extent to which m1-type muscarinic acetylcholine receptors (m1 AChRs, single-label immunoperoxidase staining profile shown in Fig. 2) are expressed by parvalbumin-immunoreactive (PV-ir, single-label immunoperoxidase staining profile shown in Fig. 3) neurons in visual areas V1 and MT of three macaque monkeys.

### m1 AChR immunoreactivity

**Figure 4:**
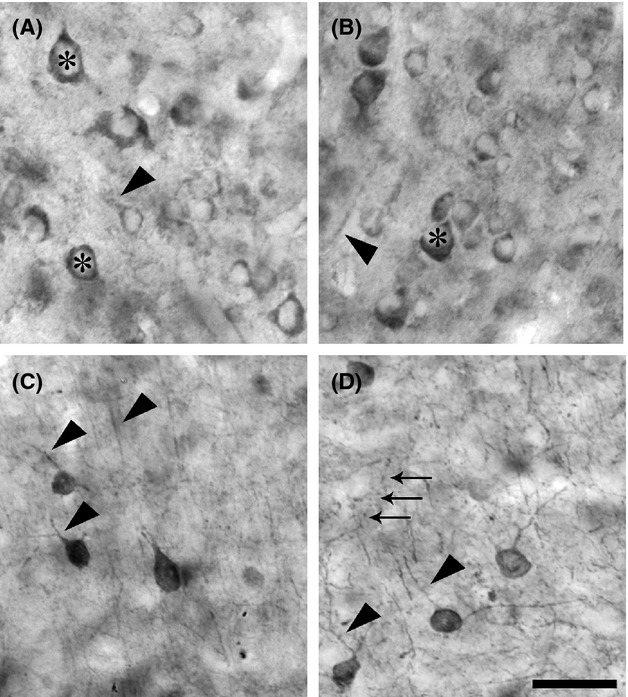
Qualitative detail of single-label immunoperoxidase reactivity for m1 ACh receptors, panels A and B) and parvalbumin (C and D) in visual areas V1 (A and C) and the middle temporal visual area (MT) (B and D). The micrograph in panel A shows m1 AChR-immunoreactivity in layer 5 of area V1. Panel B shows m1 AChR-immunoreactivity in layer 3 of MT. In both panels the characteristic stained cytoplasmic ring around an immunonegative nuclear region is evident (asterisks). When immunoreactive dendrites are evident, they are usually (arrowhead in A), but not always (arrowhead in B) visibly attached to a cell body. There was no axonal staining evident in either cortical area. Parvalbumin immunoreactivity (C and D) on the other hand, is evident in cell bodies, dendrites (arrowheads in C and D) and axons (arrows in D). Panel C is a micrograph captured in layer 2 of area V1. The parvalbumin immunoreactivity shown in Panel D is of layer 5 in MT. Scale bar = 20 *μ*m.

**Figure 5:**
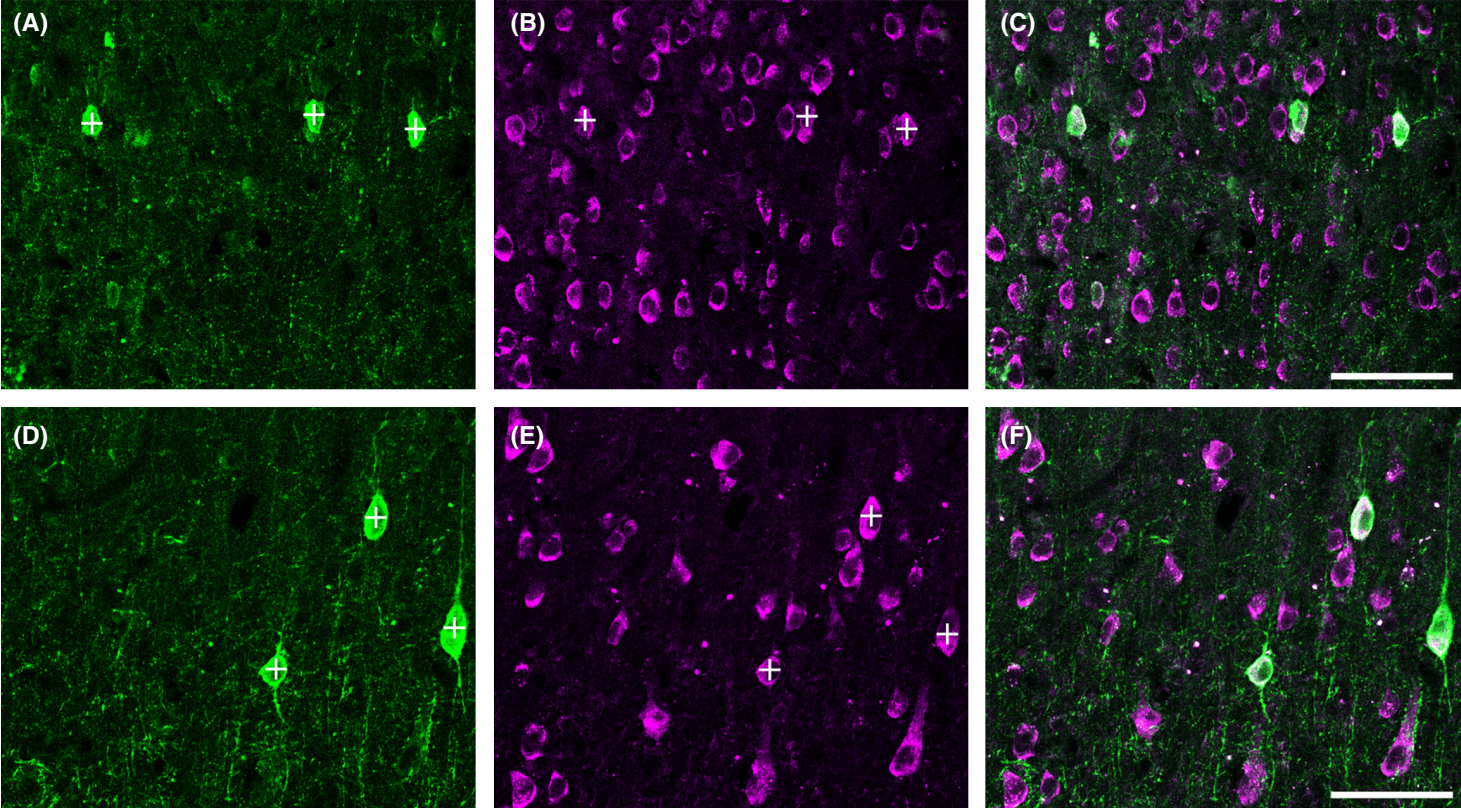
Most parvalbumin (PV) neurons (panels A and D) in both V1 (top row, A–C) and the middle temporal visual area (MT) (bottom row, D–F) express m1 AChRs (panels B and E). These images are of layer 3 from areas V1 and MT. The images were taken from the same tissue section using a 40× objective. Roughly the same size area is shown (26,148 *μ*m^2^ in V1 and 27,423 *μ*m^2^ in MT), although as a result of the higher packing density in V1, this results in a larger number of neurons imaged. The impression given, particularly comparing panels B and E, is of a larger proportion of neurons immunoreactive for m1 AChRs in V1 (B) compared with MT (E), but the packing density makes qualitative comparisons misleading. Neuropil staining is similarly strong and punctate for PV (A) and weak for m1 AChRs (B) in both areas. Most PV neurons are immunoreactive for m1 AChRs. In fact in these images all three PV neurons in V1 (+, A, B) and the three PV neurons in MT (+, D, E) are immunoreactive for m1 AChRs (note white appearance of regions of the PV somata, indicative of co-localization, in panels C and F). Scale bar = 50 *μ*m.

As reported previously, (Mrzljak et al. 1993; Tigges et al. 1997; Disney et al. 2006; Disney and Aoki 2008; Disney and Reynolds 2014) the qualitative appearance of immunoreactivity for m1 AChRs in the occipital lobe of macaque monkeys is that of a stained cytoplasmic ring around an immunonegative nuclear region (asterisks in Fig. 4A and B). This somatic pattern is accompanied by occasional labeling of the proximal dendrites (arrowhead in Fig. 4A) and some immunoreactivity of the neuropil. In both V1 and MT, m1 AChR-immunoreactive (m1 AChR-ir) somata are found in all cortical layers, including layer 1 (Fig. 2A and B). Both the somatic and neuropil immunolabel appear stronger in area MT than in V1. The stronger neuropil labeling in MT makes immunoreactive somata difficult to identify at low magnification (Fig. 2B), although they are easily identified at higher magnification and under the confocal microscope (Figs. 5, 7). Labeling of large, pyramidal-shaped somata is also more evident in MT, particularly in layer 5 (Fig. 2B, also Figs. 5, 7). This is consistent with our previously published data showing that a higher proportion of excitatory neurons in extrastriate cortex express muscarinic receptors than in the striate cortex (Disney et al. 2006). In both areas, layer 4 (4c in V1) stands out as a region of lower overall intensity of m1 AChR immunoreactivity.

### Parvalbumin immunoreactivity

The qualitative pattern of PV immunoreactivity we observe is consistent with that reported previously for parvalbumin in macaque V1 and MT (Van Brederode et al. 1990; Dhar et al. 2001; Disney and Aoki 2008). PV neurons in macaque constitute a diverse class that includes both inhibitory and excitatory neurons (Ichinohe et al. 2004; Constantinople et al. 2009). Consistent with these previous reports, we observe immunoreactive somata of diverse morphology in V1 (Fig. 4C) and in area MT (Fig. 4D). In both areas, immunolabel for PV fills the soma and much of the dendritic tree (arrowheads in Fig. 4C and D) and axonal arbor (arrows in Fig. 4D) and PV neurons are found in layers 2–6 (Fig. 3A and B). Occasionally, PV neurons are also seen in layer 1 of V1 (Fig. 3A). A higher density of PV-ir somata and processes (dendrites and axons) is evident in layers 4a, 4c (particularly the lower two thirds), and 6 of area V1 (Fig. 3A). Laminar banding is not as apparent in area MT (Fig. 3B).

### Dual m1 AChR/PV immunoreactivity

In both V1 and MT, most PV neurons are immunoreactive for m1 AChRs (Fig. 5). Other qualitative aspects of the immunolabeling are also similar when V1 and MT are compared. Although labeling intensity (both somatic and nonsomatic) in MT is slightly stronger, there is a general lack of neuropil immunoreactivity for m1 AChRs in both areas (directly compared in Fig. 5).

Quantified across 2.67 mm^2^ of macaque V1 tissue, spanning all cortical layers, eighty percent (235 of 293 raw, 164 of 205 corrected) of PV neurons express m1 AChRs, a replication of our previously published results (Disney et al. 2006; Disney and Reynolds 2014). In the same tissue sections, quantified across 4.02 mm^2^ of tissue we find that 75% (218 of 293, 153 of 205 corrected) of PV neurons in area MT express m1 AChRs. Because the correction factors were the same for m1 and PV neurons in each area (0.7) we applied the correction factor to the count of dually labeled neurons and calculated overall percentages on corrected numbers. The raw and Abercrombie corrected counts and resulting percentages collapsed across cortical layers are presented in Table 3. When calculated as a mean across the three animals in the study the results are very similar (79% dual-labeled in V1, SD 5.1 and 74% in MT, SD 0.6). Because the data do not deviate from a normal distribution (Lilliefor's test of normality, all *P* > 0.1) we use a *t* test to evaluate significance. The difference in m1 receptor expression between PV neurons in V1 and MT is not statistically significant (*P* = 0.17, two-tailed *t* test).

**Table 3:** Percentage of parvalbumin (PV)-immunoreactive neurons also immunoreactive for m1 acetylcholine receptors in V1 (top) and the middle temporal visual area (MT) (bottom)

Visual area	Raw *N* PV	Corrected *N* PV	Raw *N* dual-labeled	Corrected *N* dual-labeled	% dually-labeled
V1	293	205	235	164	80
MT	293	205	218	153	75

“Corrected” counts refer to the Abercrombie correction (See Methods).

**Figure 6:**
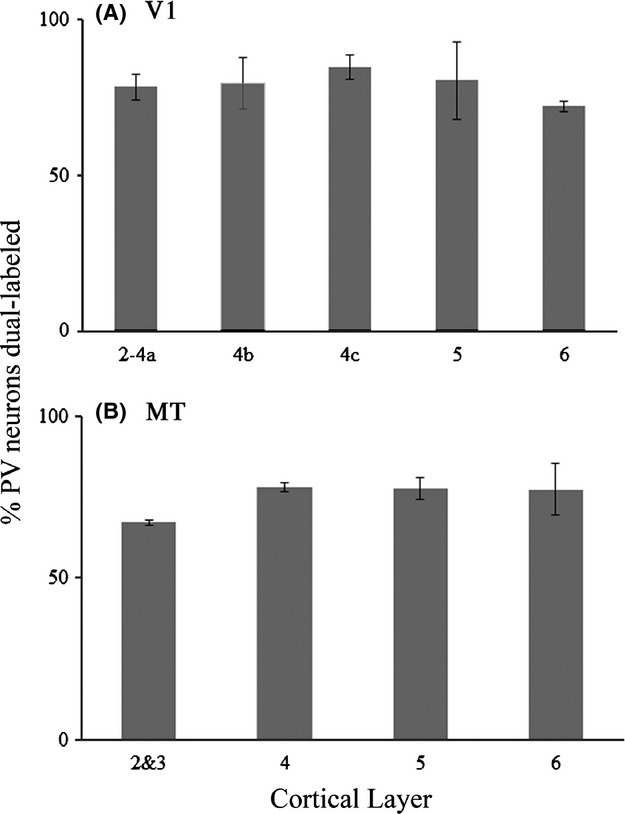
Quantification of m1 AChR expression by parvalbumin neurons. The graphs show the percentage of parvalbumin (PV) neurons encountered, by cortical layer, that were also immunoreactive for m1 AChRs in areas V1 (A) and the middle temporal visual area (MT) (B). Error bars represent SEM. Number of PV neurons in each graph is the same: V1 = 293 PV neurons, MT = 293 PV neurons.

A quantitative laminar profile of m1 AChR expression by PV neurons, comparing V1 and MT, appears in Figure 6. In both areas, the pattern of dual immunoreactivity is roughly flat across layers. In V1, the percentage of PV neurons that express m1 AChRs ranges from 73% in layer 6 to 84% in layer 4b. Percent dual labeling in area MT varies between 67% (layers 2 and 3) and 80% (layer 5). The differences between layers are not statistically significant in either V1 (*P* = 0.82, one-way analysis of variance, ANOVA) or MT (*P* = 0.22, one-way ANOVA).

**Figure 7:**
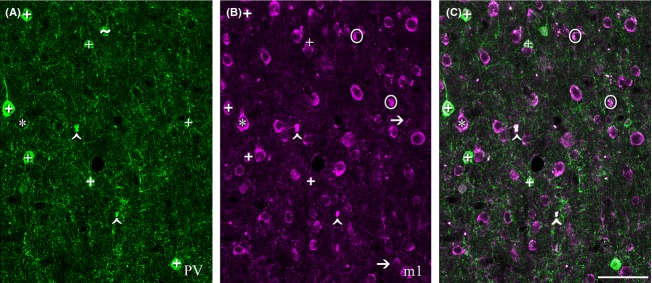
Most m1 AChR-immunoreactive neurons (panel B, magenta) in the middle temporal visual area (MT) are not immunoreactive for parvalbumin (PV; panel A, green). This image was captured in layer 5 of area MT using a 40× objective. There are seven PV neurons in the imaged field (+, A). Note that the cell body marked with a ∼ in panel A would not have been counted in this study as it does not have a clearly defined boundary within the imaging plane, although it is a PV neuron. Five of these seven PV neurons are also immunoreactive for the m1 AChR (+ in B and C). Two are m1-immunonegative (arrows in B). A striking feature of this image is the number of neurons that are immunoreactive for m1 AChRs (B, C), but not PV (A). Although these singly labeled m1-ir neurons occasionally appear pyramidal (*), it is generally not possible to determine cell morphology (or, therefore, cell class) from the immunoreactivity for m1 AChRs. The strongly fluorescent regions circled in B and C would not have been counted as they do not appear somatic in shape. These regions appear in only one channel and can thus be distinguished from the lipofuscin autofluorescence (∧ in A, B, and C) which is equally strong but appears in all channels. Scale bar = 100 *μ*m.

While most PV neurons express m1 receptors, the reverse is not true; most m1 AChR-ir neurons are not immunoreactive for PV, both in MT (Figs. 5, 7) and in V1 (Fig. 5). Forty-five percent (235 of 520 raw, 164 of 364 corrected) of V1 neurons expressing the m1 AChR are also immunoreactive for PV. In the same tissue sections, we find that 20% (218 of 1081, 153 of 757 corrected) of m1 AChR-ir neurons in area MT express PV. The raw and Abercrombie corrected counts and resulting percentages collapsed across cortical layers are presented in Table 4. When calculated as a mean across the three animals in the study PV neurons account for 45% (SD 9.6) of m1 AChR-ir neurons in V1, while in MT they account for only 20% (SD 2.7). This difference between V1 and MT is statistically significant (*P* = 0.01, two-tailed *t* test).

**Table 4:** Percentage of m1 acetylcholine receptors-expressing neurons also immunoreactive for parvalbumin in V1 (top) and middle temporal (MT) (bottom)

Visual area	Raw *N* m1	Corrected *N* m1	Raw *N* dual-labeled	Corrected *N* dual-labeled	% dually-labeled
V1	520	364	235	164	45
MT	1081	757	218	153	20

“Corrected” counts refer to the Abercrombie correction (See Methods).

**Figure 8:**
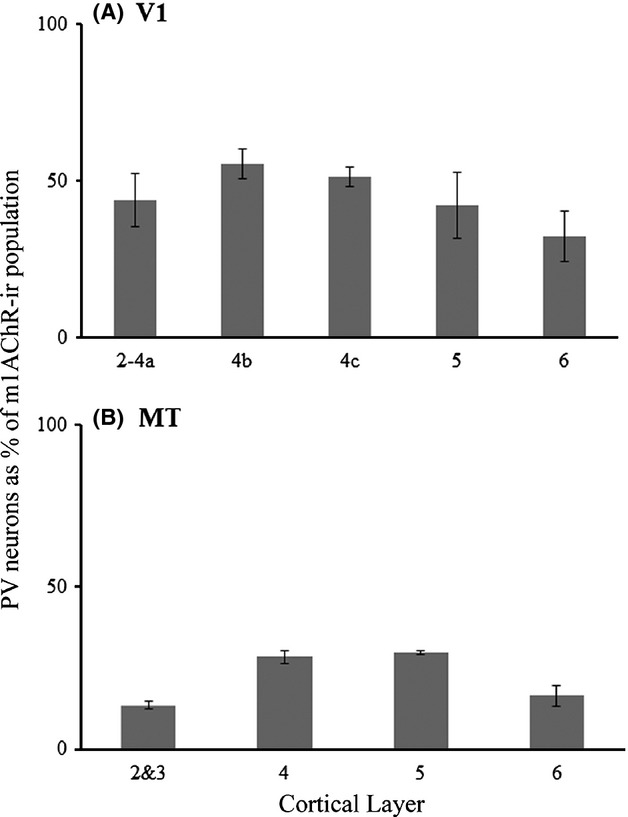
Quantification of the parvalbumin-immunoreactive population as a percentage of m1 AChR-expressing neurons. The graphs show the percentage of m1 AChR-expressing neurons encountered, by cortical layer, that were also immunoreactive for parvalbumin in areas V1 (A) and the middle temporal visual area (MT) (B). Error bars represent SEM. Number of m1 AChR-ir neurons: V1 = 520, MT = 1081.

A quantitative laminar profile of PV expression by m1 AChR-ir neurons is presented in Figure 8. In V1, the pattern of dual immunoreactivity is again very similar across layers; the percentage of m1 AChR-expressing neurons that are members of the PV-ir population ranges from 32% in layer 6 to 59% in layer 4b. These differences are not significant (*P* = 0.15, one-way ANOVA). In area MT, there is a trend toward higher m1 AChR expression in the non-PV population in layers 2/3, and 6 (Fig. 8) where the PV-ir population accounts for only 14% and 16% of the m1 AChR-expressing population respectively. PV neurons account for 29% of m1 AChR-expressing neurons in both layers 4 and 5. These laminar differences do not, however, reach significance (*P* = 0.08, one-way ANOVA).

We have previously published the expression of m1 AChRs by other classes of V1 neuron (Disney et al. 2006; Disney and Aoki 2008); m1 AChRs are expressed by 60% of calbindin-immunoreactive neurons, 40% of calretinin-immunoreactive neurons and 10% of excitatory neurons. We cannot, on the basis of the data reported here, firmly identify the neuronal classes of the non-PV neurons in area MT that express the m1 AChR. However, we also have reported previously that the proportion of excitatory neurons that express m1 AChRs is higher in the extrastriate cortex (V2) than it is in V1, (Disney et al. 2006).

**Figure 9:**
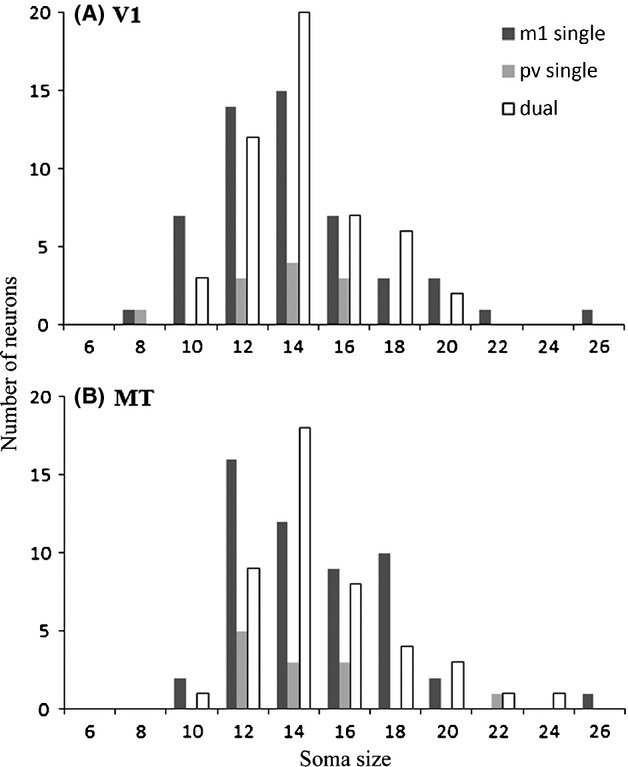
The distributions of soma sizes are similar between the populations of singly and dually labeled neurons in V1 (A) or the middle temporal visual area (MT) (B). In these histograms of soma sizes, measured along the long axis, it can be seen that in V1 (A) the distributions for singly-labeled parvalbumin (PV)- and m1 AChR-ir neurons, and for dually immunoreactive neurons are unimodal and centered around 12–14 microns. In MT these distributions are shifted to the right and somewhat skewed, with a long tail at the end representing soma sizes greater than 20 *μ*m. This tail of the distribution has a very small *N*, but all three groups (single PV-ir, single m1AChR-ir and dual PV/m1-ir) are represented in the population of neurons with large somata, offering no evidence for a bi-modal distribution of soma size in either cortical area. *N* for V1: m1 single = 52, PV single = 11, dual = 50 neurons. *N* for MT: m1 single = 52, PV single = 12, dual = 45 neurons.

One indicator that the singly labeled m1 AChR-expressing neurons (i.e., those that are not PV-ir) come from a different neuronal class would be differences in soma size. However, the PV-ir and the PV-immunonegative subpopulations of m1 AChR-expressing neurons in both areas have similar soma sizes. The mean soma size for singly labeled m1 AChR-ir neurons in V1 is 13.04 *μ*m (SD 3.3) and the mean for dually labeled neurons is 13.22 *μ*m (SD 2.59). The mean soma size for singly labeled m1 AChR-ir neurons in area MT is 13.86 *μ*m (SD 3.10) and the mean for dually labeled neurons is 13.94 *μ*m (SD 2.93). The soma size distributions are also similar (Fig. 9). These distributions all deviate from normality (Lilliefors test) and so we use the nonparametric Mann–Whitney *U* statistic to evaluate differences between means. None of the differences in soma size between the singly-labeled (PV or m1 AChR) and dually-labeled populations in either cortical area was significant (*P* > 0.05).

Directly comparing the m1 immunoreactivity profiles in V1 and MT, without other labels to identify specific neuronal classes, has little benefit; both because individual cell morphology is not evident from the m1 AChR immunolabeling and because differences in neuropil composition, packing density, soma size, and cortical thickness (Rockel et al. 1980; Hendry et al. 1987; Beaulieu et al. 1992; Carlo and Stevens 2013) can give qualitative impressions that are misleading. However, based on sheer numbers (because the majority of neurons in any cortical area are excitatory) it seems likely that excitatory neurons make up the vast majority of non-PV, m1 AChR-expressing neurons in area MT.

## Discussion

In this study, we report that most parvalbumin-immunoreactive (PV) neurons in both visual areas V1 and MT of macaque cortex, express m1-type muscarinic acetylcholine receptors (m1 AChRs). Specifically, m1 AChRs are expressed by 80% of PV neurons in area V1 and 75% of PV neurons in area MT. We also report that PV neurons comprise a smaller proportion of m1 AChR-expressing neurons in area MT (20%) than in area V1 (45%). It is important to note that while we report the area of the tissue examined, and offer an Abercrombie correction for all counts made, the data in this study were not collected using stereological methods and should not, therefore, be used to calculate total numbers or densities of neuronal types for either V1 or MT.

### PV neurons as targets for cholinergic neuromodulation

Parvalbumin (PV) neurons are a heterogeneous population that includes two well-studied interneuron subtypes: large basket and chandelier cells. DeFelipe et al. (1999) report that there are very few chandelier cells in V1 (these cells are more common in the extrastriate visual areas), and that in V1 they appear to be largely restricted to layer 2. However, PV-immunoreactive (PV-ir) basket cells are found in all layers of V1 (Van Brederode et al. 1990; DeFelipe et al. 1999). Basket cells have sparsely branched axons, which give off small perisomatic, basket-shaped amplifications at intervals along their length. Chandelier cells make synapses in arrays along the axon initial segment of their target neurons. Both of these cell types thus make synapses at locations which allow control over a target cells' firing rate or pattern (or both). The current data, combined with a previous study showing that iontophoresis of ACh increases GABA release in macaque V1 (Disney et al. 2012) suggest that increases in inhibitory tone during ACh release could be expected in MT.

A proposed function of perisomatic inhibition is the control of spike timing and generation of synchronous spiking across populations of principal cells (Freund 2003). Neuromodulators, including ACh, can alter the strength and frequency of oscillations, with particularly strong effects in the *γ* band (Giocomo and Hasselmo 2007). Our finding that neurons providing perisomatic inhibition in both V1 and MT express m1 AChRs, would lead to the prediction that ACh will modulate the level of synchronous firing, probably in the *γ* band, in both cortical areas.

### Non-PV neurons as targets for cholinergic modulation

We were unable, on the basis of the data gathered in this study, to definitively identify the neuronal class of the large number of non-PV neurons in MT that express the m1 AChR. However based on their sheer numbers (80% of m1 AChR-ir neurons were not PV-ir), our prior report that the proportion of excitatory neurons that express m1 AChRs is higher in the extrastriate cortex (V2) than it is in V1, (Disney et al. 2006), and our unpubl. obs. (A. A. Disney) that a little more than half of the m1 AChR-expressing population in area MT is not immunoreactive for GABA; this group of cells is likely to be largely comprised of excitatory neurons.

### Acetylcholine and attention

It has been proposed that ACh plays a role in attention (Everitt and Robbins 1997; Sarter et al. 2005). Most – but not all (Herrero et al. 2008) – of the research implicating ACh release in attention has been conducted in rodents. At the same time our most sophisticated behavioral models of attention arise from research in macaque monkeys. What role does ACh play in attention in the macaque? Attention has been shown to alter the gain of neuronal responses throughout the visual pathway of macaques (Motter 1993; Treue and Maunsell 1996; McAdams and Maunsell 1999; Reynolds et al. 2000). ACh has also been found to multiplicatively increase the gain of the ascending visual input to V1 of the macaque (Disney et al. 2007). Similarly, attention has been shown to suppress activity in response to task-irrelevant distracters (Moran and Desimone 1985; Reynolds et al. 1999; Sundberg et al. 2009) and to alter the frequency of oscillatory activity in cortical networks (Fries et al. 2001; Chalk et al. 2010). Both of these functions could be enabled through the control of soma-targeting and lateral inhibition.

Attention has also been shown to strongly and consistently influence the spiking behavior of narrow-spiking, nonbursting neurons in area V4 (Anderson et al. 2011a) a population that is likely to be largely made up of PV neurons (Kawaguchi and Kubota 1993; Chow et al. 1999; Constantinople et al. 2009; Anderson et al. 2011a). The overall effects of attention on this population of putatively inhibitory neurons are stronger and more consistent than the overall effects of attention on their excitatory neighbors (Mitchell et al. 2007). The common model for attention effects in area V4 proposes that glutamatergic feedback from the frontal eye fields carries a modulating attention signal to V4. These feedback projections are known to preferentially target excitatory neurons (Anderson et al. 2011b). For a “glutamatergic feedback cascade” model to hold, we need to explain an effect on inhibitory neurons that is generally stronger and more consistent than the effect observed in the population of cells that drives them. One solution is to propose amplifying local connectivity and/or a specific feedback-receptive sub-circuit and to then seek evidence of the necessary supporting neural architectures. An alternate (not in fact mutually exclusive) explanation envisages post-synaptic amplification of the feedback signal by a neuromodulatory system acting through specific and strong receptor expression by PV neurons.

Such a model is consistent with the strong expression of m1 AChRs by PV neurons that we report here. But we also report strong expression by non-PV neurons, many of which are likely to be excitatory. If many excitatory neurons show weak and inconsistent effects of attention and many inhibitory neurons show strong and consistent effects of attention – can this possibly be mediated by ACh acting via a receptor type (m1) that is, expressed in both cell classes?

Anderson et al. (2011a) report that there is a population of putatively excitatory neurons that do show strong modulation of firing rate by attention – neurons that fire their spikes in bursts. If ACh is the mediator of attention-related effects in extrastriate cortex, then one prediction from the current data would be that both narrow-spiking neurons and broad-spiking neurons that tend to fire their spikes in bursts should be sensitive to m1 AChR-selective agonists and antagonists while those that do not fire spikes in burst will be insensitive to these same pharmacological agents.

### Downstream targets for m1 AChR-mediated modulation

It could also be that the downstream targets for m1 AChRs differ in the two classes of cells – leading to different forms and strengths of cholinergic modulation. Predicting the functional effect of activating anatomically identified muscarinic receptors is challenging because they are coupled to G-proteins and thus to complex and diverse intracellular signaling cascades. Excitatory neurons are a well-known target for cholinergic modulation acting through the m1 AChR. Acetylcholine binding to this receptor can transiently close the m-type potassium current (K_m_) leading to a decrease in spike frequency adaptation and an increase in bursting (Brown et al. 1997; Yue and Yaari 2004) which probably changes these cells' participation in local oscillatory dynamics (Fuhrmann et al. 2002). This current is likely a major contributor to cholinergic effects on m1 AChR-expressing excitatory neurons.

Many PV neurons, however, (specifically those that comprise the population of fast-spiking inhibitory neurons) do not express an m-current. These are neurons that fire at high rates *without significant adaptation*. Thus the principal target for m1 AChR-mediated effects on these neurons is less obvious. Usually in vitro studies would have elucidated the effects of a common receptor like the m1 AChR on a major neuronal class such as the PV neurons, but PV neurons in rats and mice (the species most often used in in vitro studies) do not appear to express muscarinic receptors very strongly, if at all (Kawaguchi 1997; Gulledge et al. 2007; Disney and Reynolds 2014). In an early study, McCormick and Prince reported that ACh, acting via a muscarinic receptors, does excite a class of neurons in the guinea pig cingulate cortex that emit narrow spikes (McCormick and Prince 1986) and most PV neurons in guinea pig cortex do express m1 AChRs (Disney and Reynolds 2014). Thus, it seems likely that ACh could excite PV neurons via m1 AChRs. We have shown previously that whatever the mediating receptor or downstream effector, ACh released into V1 induces the release of GABA, indicating that ACh can excite inhibitory neurons in V1 (Disney et al. 2012), as would be required if cholinergic modulation were the underlying cause of attention-mediated increases in spike rate in narrow-spiking neurons.

Most of the work on cholinergic modulation in the macaque has been undertaken in area V1, although it has recently been shown that local ACh release changes a number of response properties in MT (Thiele et al. 2012). Very little is known about AChR expression, release or local effects in areas – such as MT or V4 – where neuronal responses are more strongly modulated by attention. The present study suggests that experiments conducted on cholinergic control of inhibition in V1 may well be broadly applicable across the visual cortex. Our finding that expression of m1 AChRs by excitatory neurons is stronger in MT than in V1, combined with the similar expression by PV neurons makes it likely that other features of cholinergic modulation of the cortical circuit and particularly of the balance of excitation and inhibition will change as one moves up through the visual pathway.

### Other classes of muscarinic receptors

In this study, we looked at one type of muscarinic AChR, type m1. There are four other types of muscarinic AChRs; m2-5. Previous in situ hybridization studies of the macaque cortex suggest that receptor types m3 and m4 are not strongly expressed in the visual cortex (Tigges et al. 1997). Unfortunately, antibodies directed against m3, m4 and m5 AChRs do not pass controls for use in the macaque monkey (Disney and Reynolds 2014; and A. A. Disney unpubl. data) so these anatomical data are unlikely to become available in the near future. Some antibodies against the m2 AChR do pass controls in macaques (Disney et al. 2006, 2007; Disney and Aoki 2008). This receptor type is also expressed by many PV neurons in macaque V1 (Disney and Aoki 2008). A future study of m2 expression in extrastriate cortical areas would be valuable. Such a study was beyond the scope of the present investigation as it would need to include electron microscope (EM)-level data collection, because the m2 AChR is frequently trafficked out of the cell body into the axon (Disney et al. 2006). In this case it needs to be confirmed, by EM, that counting somata does not underestimate the m2-immunoreactive population.

Also of interest in the future would be a study of AChR expression by calbindin- and calretinin-immunoreactive cells in MT. These are critical populations to describe for the extrastriate cortex as the proportion of inhibitory cells that express calbindin and calretinin increases as one moves up through the visual pathway (DeFelipe et al. 1999) and into the prefrontal cortex (Conde et al. 1994). In V1, these classes comprise fewer than 5% of all neurons (Hendry et al. 1990; Van Brederode et al. 1990; Beaulieu et al. 1992; Meskenaite 1997). Due to their low density, different tissue sampling methods would have been required in order to include a comparison of these populations between MT and V1 in the present study.

In conclusion, PV neurons in visual areas V1 and MT frequently express m1 AChRs, and in fact the majority of this class of neurons in both areas are m1 AChR immunoreactive. These data support the use of V1 as a model circuit for cholinergic modulation of PV neuron-mediated inhibition in the visual cortex. They also provide a possible mechanism for the particularly strong effects of attention on narrow-spiking neurons in the extrastriate cortex.
